# A Cross-Sectional Study Investigating the Knowledge and Attitude of Health Professions Students in Saudi Arabia: Are They Ready for Cardiopulmonary Resuscitation?

**DOI:** 10.7759/cureus.43048

**Published:** 2023-08-06

**Authors:** Mohammed Awawdeh, Abdullah M Alanzi, Meshal Alhasoun, Abdulilah Babtain, Nasser Alshahrani, Ahmed Alhamdan, Naif Almutairi, Alaa Oteir, Khader Almhdawi

**Affiliations:** 1 Preventive Dental Science, College of Dentistry, King Saud bin Abdulaziz University for Health Sciences, Riyadh, SAU; 2 Dentistry, College of Dentistry, King Saud bin Abdulaziz University for Health Sciences, Riyadh, SAU; 3 Allied Medical Sciences, Jordan University of Science and Technology, Irbid, JOR; 4 Rehabilitation Sciences, Jordan University of Science and Technology, Irbid, JOR

**Keywords:** chest compression, life-saving skill, cardiac arrest, emergency training, external defibrillators, medical training, saudi arabia, students, cpr attitude, cpr knowledge

## Abstract

Introduction: Cardiopulmonary resuscitation (CPR) training is important for students of health professions to learn and be prepared to perform. Colleges have a responsibility to provide adequate training for their students to ensure that they are ready and confident to deal with life-threatening situations. However, studies have shown that some graduates and practitioners lack sufficient knowledge in performing CPR. The aim of this study is to assess the knowledge of health professions students in the Kingdom of Saudi Arabia (KSA) who have started clinical practice.

Methodology: This cross-sectional study was conducted in February 2022 and included health professions students in all academic years, including interns and residents, across colleges of King Saud bin Abdulaziz University for Health Sciences, Riyadh, KSA. The study questionnaire consisted of three sections: attitudes, knowledge, and demographics. The attitudes section included 11 questions, while the knowledge section included 10. The demographic section included university level, Grade Point Average (GPA), CPR training status, willingness to learn CPR, witnessing CPR, and family history of cardiac disease. Statistical analysis was conducted using chi-squared tests, t-tests, two-sample proportion tests, ANOVA, and bivariate correlation analyses.

Results: The mean age of the participants was 21.2 (±1.9) years. Participants had a mean knowledge score of 5.1 (±1.8) out of 10 potential points. Also, the participants had a total attitude score of 42.7 (±6.2) out of 55 potential points.

Conclusion: The study highlights the importance of CPR training for healthcare providers and the need for ongoing training to maintain knowledge and skills. The results suggest that attitudes towards providing CPR may be influenced by cultural beliefs and fear of liability or disease transmission. Higher participant GPA and positive attitudes towards chest compressions and CPR training were found to be associated with increased knowledge.

## Introduction

Cardiopulmonary resuscitation (CPR) is a life-saving procedure that is performed for cardiac arrest and includes chest compressions with or without ventilation [1]. It is crucial for all health professions students to be ready and equipped to deal with medical emergencies and perform CPR when and where needed. CPR training could be the difference between a patient who survives and another who does not when an emergency takes place in the clinic. The training and availability of automatic external defibrillators (AED) are also important in the immediate management of cardiac arrest patients [2].

Acquiring sufficient knowledge and understanding of emergency measures in life-threatening situations improves the confidence of the practitioner and, consequently, might improve the ability to act properly in such situations [3]. For this reason, colleges in the medical, nursing, and dental fields have a great responsibility to provide comprehensive knowledge and training that a graduating health practitioner or professional should have [2,3]. Having adequate knowledge of the appropriate way to deal with a patient who is having a cardiac arrest renders the clinician capable of making decisions with confidence, which results in the patient feeling that he/she is being treated by an experienced, professional practitioner [4]. Moreover, the inclusion of CPR early in the curriculum increases health professions students’ appreciation and awareness of this life-saving skill [5]. Practitioners should expect to encounter medical emergencies at any time during their practice, and the best approach to deal with such situations competently is to learn such skills in advance [6].

In a cross-sectional study carried out in Germany, 57% of the responders, who were dentists, reported experiencing up to three emergencies within one year [7]. As a general rule, a plan to appropriately manage emergency events should include dental staff training, construction and publication of emergency-related guidelines, and provision of knowledge and training on the utilization of the emergency kit [3]. Although schools in the medical field have put an effort to deliver the necessary knowledge about the management of different types of medical emergencies, dental graduates and practitioners still show a significant amount of deficiency in knowledge regarding emergency situations [6]. The defects have been identified and reported in numerous studies; for example, Jodalli and Ankola reported a low level of knowledge about emergencies, related drugs, and equipment among dental interns in 2010 in Belgaum, India [6]. In this study, the participants disclosed that medical emergencies should be an essential topic in the study plan of dentistry. Moreover, most of the participants expressed that they were willing to be trained in important courses related to medical emergencies [6].

Another study, in Indiana, highlighted an apparent confusion among dental residents and periodontists in different degrees of misdiagnoses and the handling of multiple medical emergency cases [5]. This study, along with other studies, concluded the need for more training about case-specific features for dental faculty and residents. In a survey that was done on Saudi dental undergraduates and interns, it was concluded that multiple factors played a major role in the reduction of mortality rate related to medical emergencies in the dental chair [8]. The factors are as follows: availability of medications, availability of basic emergency equipment, and well-trained dental staff. This study, however, was limited by the fact that it only measured the effectiveness of CPR training in the dental curriculum. It is known that students enrolled in other specialties like medicine and emergency medical technicians undergo more training when compared to dentistry in terms of medical emergencies. In another study that was done on undergraduate dental students and practitioners at the Taibah Dental College, KSA, it was demonstrated that many participants showed a moderate level of knowledge in regard to the management of medical emergencies [9]. For example, when participants were questioned about the appropriate way to manage “crushed chest pain”, only 54% responded correctly. Overall, participants scored 50-74% correct responses regarding crushed chest pain, sudden onset of brain stroke, psychiatric patients, unconscious patients with hypoglycemia, patients with postural hypotension, and patients with hyperventilation [9]. This study was limited by the low sample size (202 students, 41 trainers), and not using validated standardized outcome measures. Also, 78% of trainers were non-Saudis, which indicated a different background in terms of medical emergency training.

The need for effective training of practitioners to handle medical emergencies has been well documented by many researchers [10]. When designing a medical curriculum, it is important that the quality of training to handle medical emergencies be improved to enhance health professions students’ ability to recognize, manage, or request professionals to deal with emergency cases [10]. In KSA, it is a prerequisite in all health university programs that health practitioners attend a course that includes didactic knowledge and hands-on training in cardiopulmonary resuscitation and pass an exam before starting the clinical part of the curriculum [8]. Such a measure is a sign of good practice that improves patients’ welfare and protects practitioners from legal encounters. Therefore, the aim of this study is to assess the knowledge and attitude of health professions students in KSA who have started clinical practice regarding CPR. Second, the study aims to compare CPR-related knowledge and attitudes between gender and CPR training status. Finally, the study assesses CPR knowledge and attitude correlates. This data will be helpful to understand the current level of knowledge and attitude in health universities so that regulatory institutions can adjust the curricula accordingly. We hypothesize that there is a significant difference in knowledge and attitude of the King Saud bin Abdulaziz University for Health Sciences (KSAU-HS) health professions students toward CPR between different CPR training levels, gender, and Grade Point Average (GPA).

## Materials and methods

This cross-sectional study was conducted in KSAU-HS in KSA targeting the health professions students of all majors. The study was conducted between February 2022 and June 2023 and targeted male and female students in all academic years, including interns and residents, across colleges in KSAU-HS. The Institutional Review Board of King Abdullah International Medical Research Center approved the study (protocol number: NRC22R/072/02).

Study questionnaire

The study questionnaire was adapted from two previously published studies [11,12]; In these studies, the questionnaire was proven valid and reliable through a multi-step validation system which included students and an expert panel of professionals The expert panel approved a final version of the survey. A multidisciplinary expert group, including academics, developed the questionnaire by utilizing the American Heart Association (AHA) guidelines, as well as previous studies and clinical and academic experience. The expert panel also assessed the questionnaire for face and content validity. A second group consisting of four paramedicine professionals evaluated the questionnaire providing feedback that further improved the survey’s validity. The questionnaire was then piloted to a group of 30 applied health profession students.

The study questionnaire included three sections: attitudes, knowledge, and demographics. The attitudes section consisted of 11 questions divided into three subsections, including attitudes toward CPR in general as well as toward performing mouth-to-mouth ventilation (MMV) and chest compressions to different groups such as strangers, relatives, and children, and included concern about infection. Each of the 11 questions had a five-point Likert scale, where a higher score indicates a more positive attitude. To simplify the interpretation to the participants, the final attitudes scale scores were categorized into (yes) ‘scores of 4 and 5’, (neutral) ‘score of 3’, and (no) ‘scores of 1 and 2’. The knowledge section consisted of 10 questions evaluating the knowledge about performing CPR and a question about the emergency phone number in Saudi Arabia. Nine of these questions had four different potential answers with only one correct answer, and one question was a true/false question. The scores in this section ranged from 0 to 10, where 10 indicated that all the questions were answered correctly. The last section included participants’ demographic information including university level, university cumulative GPA (out of 5 points), the status of previous CPR training (whether the participant has had any professional training in the past), willingness to learn CPR, whether they had witnessed CPR in progress, and whether they have family members with a history of cardiac disease. In the current study, trained individuals were defined as students who had CPR certification or hands-on training with post-training summative assessment by professionals.

Statistical analysis

Continuous variables were presented as means and standard deviation, while categorical variables were presented as counts and proportions. For attitudes, differences between groups were compared utilizing chi-squared tests for categorical variables and independent t-tests for continuous variables. Two-sample proportion tests were used to examine the differences between participants that had positive attitudes about chest compressions and MMV. A p-value of 0.05 was considered statistical significance. For knowledge, ANOVA statistical tests were used to compare means between groups. Bivariate correlation analyses were used to explore the factors associated with CPR knowledge and attitudes scores.

## Results

A sample of 314 university students participated in the study. As shown in Table 1, the majority of the sample (75.8%) were males, and 54.8% declared receiving CPR training. The mean age of the participants was 21.2 (±1.9) years. Participants had a mean knowledge score of 5.1 (1.8) out of 10 potential points. Also, the participants had a total attitude score of 42.7 (±6.2) out of 55 potential points. Furthermore, the participants had subscores of 16.9 (±2.7), 13.1 (±3.8), and 8.8 (±1.5) in the attitude towards chest compression, attitude towards MMV, and CPR importance subdomains, respectively.

**Table 1:** Participant demographics and summary of outcome measures MMV: Mouth-to-Mouth Ventilation; GPA: Grade Point Average; CPR: Cardiopulmonary Resuscitation

Variable		Values (n=314)
Age, mean (SD)		21.2 (±1.9)
GPA, mean (SD)		4.3 (±0.6)
Gender	Female, n (%)	76 (24.2%)
	Male, n (%)	238 (75.8%)
Trained	No, n (%)	142 (45.2%)
	Yes, n (%)	172 (54.8%)
Total knowledge score, mean (SD)		5.1 (±1.8)
Total attitude score, mean (SD)		42.7 ± (6.4)
Attitudes towards chest compression, mean (SD)		16.9 (±2.7)
Attitudes towards MMV, mean (SD)		13.1 (±3.8)
CPR importance, mean (SD)		8.8 (±1.5)

Table 2 discusses the difference in knowledge and attitude between trained and untrained participants. No statistically significant difference was observed in knowledge or attitude based on training status.

**Table 2:** Knowledge and attitude difference between trained and untrained participants CPR: Cardiopulmonary Resuscitation; MMV: Mouth-to-Mouth Ventilation

	CPR training	N	Mean	SD	p-value
Total knowledge	No	142	4.8	1.6	0.09
	Yes	172	5.3	1.8	
Total attitude score	No	142	41.9	6.2	0.64
	Yes	172	43.3	6.5	
Attitude toward MMV	No	142	13.1	3.6	0.18
	Yes	172	13.1	4.0	
Attitude toward chest compression	No	142	16.5	2.8	0.26
	Yes	172	17.2	2.7	
Attitude toward CPR	No	142	8.7	1.6	0.06
	Yes	172	8.9	1.4	

Table 3 discusses the difference in knowledge and attitude between male and female participants. No statistically significant difference was observed in knowledge or attitude based on gender.

**Table 3:** Knowledge and attitude differences between males and females participants CPR: Cardiopulmonary Resuscitation; MMV: Mouth-to-Mouth Ventilation

	Gender	N	Mean	SD	p-value
Total knowledge	Female	76	5.3	1.8	0.26
	Male	238	5.0	1.7	
Total attitude score	Female	76	42.4	5.8	0.62
	Male	238	42.8	6.6	
Attitudes toward MMV	Female	76	12.1	3.8	0.71
	Male	238	13.4	3.8	
Attitude toward chest compression	Female	76	17.4	2.6	0.47
	Male	238	16.7	2.8	
Attitude toward CPR	Female	76	8.8	1.6	0.78
	Male	238	8.9	1.5	

Knowledge responses

As shown in Table 4, over half of the respondents knew the correct first step when sighting an adult laying on the floor. Moreover, the majority knew the first step when performing CPR as well as the ventilation ratio. When it came to the compression number per minute, however, only 29% chose the correct answer. In regard to the questions about chest compression depth and maximum time until CPR is started, 31.5% and 32.8% chose the correct answer, respectively. Lastly, the majority of participants knew the emergency number to call in case of a medical emergency.

**Table 4:** Students’ responses to the knowledge questions Correct answers are in bold. CPR: Cardiopulmonary Resuscitation

Item	Answers	N	%
You were alone and sighted an adult laying on the floor, what would be the most important step to do?	Call for help or emergency number	71	22.6
	Start compressions immediately	16	5.1
	Check pulse	53	16.9
	Check consciousness and breathing	174	55.4
Which of the following is true regarding CPR?	Giving mouth-to-mouth ventilation is more important and superior to chest compression	6	1.9
	CPR starts with mouth-to-mouth ventilation and chest compressions simultaneously	48	15.3
	CPR starts with mouth-to-mouth ventilation	32	10.2
	CPR starts with chest compressions	228	72.6
What is the compression-to-ventilation ratio for an adult patient?	15 compressions: 1 ventilation	28	8.9
	5 compressions: 1 ventilation	20	6.4
	30 compressions: 2 ventilations	206	65.6
	30 compressions: 5 ventilations	60	19.1
What is the number of compressions per minute for an adult patient?	100-120 compressions per minute	92	29.3
	60-80 compressions per minute	119	37.9
	80-100 compressions per minute	73	23.2
	More than 120 compressions per minute.	30	9.6
	Compressing slowly	10	3.2
Which of the following is a characteristic of true effective CPR?	Allowing full chest recoil after each compression	228	72.6
	Compressing fast but not hard	52	16.6
	Compression without allowing chest recoil	24	7.6
What is the depth of compression for an adult patient?	At least 6 cm	15	4.8
	5 to 6 cm	99	31.5
	3 to 4 cm	119	37.9
	2 to 3 cm	81	25.8
Once the need for CPR is confirmed, chest compressions should start within a maximum of	30 seconds	55	17.5
	15 seconds	40	12.7
	10 seconds	103	32.8
	5 seconds	116	36.9
Which of the following is a characteristic of true effective CPR?	Pushing (compressing) with medium power	61	19.4
	Pushing (compressing) slowly	15	4.8
	Pushing (compressing) with medium speed	100	31.8
	Pushing (compressing) hard and fast	138	43.9
What is the medical emergency number in Saudi Arabia?	I don't know the number	43	13.7
	999	17	5.4
	998	28	8.9
	997	210	66.9
	996	16	5.1
Sudden loss of consciousness may indicate the need for CPR	Yes	219	69.7
	No	69.7	30.3

Attitude responses

As shown in Table 5, according to the score categories described in the study questionnaire section, participants were neutral to providing MMV for strangers or the other gender. Moreover, participants were willing to provide chest compressions for relatives and strangers, although they also scored "yes" to the question about being fearful of getting an infection. Lastly, participants expressed that they believe that CPR is important and are willing to receive more training in it.

**Table 5:** Mean score of attitude questions COVID-19: Coronavirus Disease 2019

Questions	Mean	SD
I would provide mouth-to-mouth ventilation to strangers	3.13	1.331
I would provide mouth-to-mouth ventilation to relatives	3.89	1.139
I would provide mouth to mouth-to-mouth mouth ventilation to the other gender	3.29	1.277
I avoid providing mouth-to-mouth ventilation due to fear of infections (Such as COVID-19)	2.77	1.335
I would provide mouth-to-mouth ventilation to children	3.78	1.243
I avoid providing chest compression due to fear of infections	4.11	1.207
I would provide chest compression to relatives	4.51	.912
I would provide chest compression to strangers	4.49	.858
I believe that cardiopulmonary resuscitation is harmful	3.87	1.337
I would like to learn and practice cardiopulmonary resuscitation encouraged by cultural values and religious beliefs	4.20	1.050
I believe that cardiopulmonary resuscitation is important and can increase the patient survival	4.63	0.794

## Discussion

This study assessed health professions students in KSA who have started clinical practice regarding CPR. In our study, three main variables were tested which are the effects of CPR training, gender, and GPA on knowledge and attitude towards CPR.

Firstly, receiving training was proposed to be a key factor in increasing confidence and improving attitude to provide CPR to those in need [13-15]. A study conducted at King Khalid University, Abha, KSA, stated that the level of education has a significant impact on knowledge, while gender has no significant impact on it [16]. However, according to our results, there was no statistically significant difference between those who were trained and those who were not. This contradiction may be correlated to the necessity of retaking CPR courses by those who showed sub-optimal results. In previously published studies, gender differences demonstrated some influences in regard to attitude in providing CPR [17-18]. These influences can be observed especially when dealing with the opposite gender [19-23]. Hesitation to provide chest compression or MMV to the other gender may be linked to either cultural beliefs, fear of being legally liable, or even fear of disease transmission [19-23]. Nevertheless, gender showed no statistically significant difference. Finally, in our study, a higher GPA was correlated with a more positive attitude toward CPR.

The results of this study shed light on the importance of being trained in CPR to be able to control emergencies that could be encountered by healthcare providers [23]. Those who had previous training on CPR during the past two years, whether it was self-studied or by professionals, have demonstrated significantly better knowledge and attitude towards CPR than their counterparts who had not received any training. However, the study's limited sample size, sampling bias (only one university included), the fact that measures were self-reported, and lack of consideration for other influential factors, including cultural background, pose potential limitations to the findings' generalizability and depth. Further studies that address our limitations and provide valuable insights on participants' Basic Life Support (BLS)/Advanced Cardiovascular Life Support (ACLS) certification status as well as whether participants are students, interns, or residents would provide more accurate data and conclusions.

## Conclusions

The result of our study demonstrates that there are no statistically significant differences in knowledge and attitude when comparing trained participants with untrained ones or when comparing both genders. However, further analysis of knowledge was found to be correlated with increased participant GPA, positive attitude about providing chest compressions, and CPR training. Overall, in knowledge questions, most participants chose the correct answer in diagnosis and initial assessment but showed lower levels of knowledge in questions related to the technical aspects of CPR. Moreover, participants showed a positive attitude towards giving CPR and expressed willingness to receive further CPR education and training.

## References

[1] Sreevastava DK, Roy PK, Dass SK, Bhargava A, Chakrabarty A, Rai V, Tarneja VK (2004). Cardio-pulmonary resuscitation : an overview of recent advances in concepts and practices. Med J Armed Forces India.

[2] Cummins RO, Eisenberg MS, Bergner L, Hallstrom A, Hearne T, Murray JA (1984). Automatic external defibrillation: evaluations of its role in the home and in emergency medical services. Ann Emerg Med.

[3] Vaughan M, Park A, Sholapurkar A, Esterman A (2018). Medical emergencies in dental practice - management requirements and international practitioner proficiency. A scoping review. Aust Dent J.

[4] Smereka J, Aluchna M, Aluchna A, Szarpak Ł (2019). Preparedness and attitudes towards medical emergencies in the dental office among Polish dentists. Int Dent J.

[5] de Bedout T, Kramer K, Blanchard S, Hamada Y, Eckert GJ, Maupome G, John V (2018). Assessing the medical emergency preparedness of dental faculty, residents, and practicing periodontists: an exploratory study. J Dent Educ.

[6] Jodalli PS, Ankola AV (2012). Evaluation of knowledge, experience and perceptions about medical emergencies amongst dental graduates (interns) of Belgaum City, India. J Clin Exp Dent.

[7] Van de Velde S, Roex A, Vangronsveld K (2013). Can training improve laypersons helping behaviour in first aid? A randomised controlled deception trial. Emerg Med J.

[8] Al-Shamiri HM, Al-Maweri SA, Shugaa-Addin B, Alaizari NA, Hunaish A (2017). Awareness of basic life support among Saudi dental students and interns. Eur J Dent.

[9] Gazal G, Aljohani H, Al-Samadani KH, Nassani MZ (2021). Measuring the level of medical-emergency-related knowledge among senior dental students and clinical trainers. Int J Environ Res Public Health.

[10] Al-Samadani KH, Gazal G (2015). Effectiveness of benzocaine in reducing deep cavity restoration and post-extraction stress in dental patients. Saudi Med J.

[11] Oteir AO, Almhdawi KA, Kanaan SF, Alwidyan MT, Williams B (2019). Cardiopulmonary resuscitation level of knowledge among allied health university students in Jordan: a cross-sectional study. BMJ Open.

[12] Oteir AO, Kanaan SF, Alwidyan MT, Almhdawi KA, Williams B (2021). Validity and reliability of a cardiopulmonary resuscitation attitudes questionnaire among allied health profession students. Open Access Emerg Med.

[13] Urban J, Thode H, Stapleton E, Singer AJ (2013). Current knowledge of and willingness to perform hands-only CPR in laypersons. Resuscitation.

[14] Berdowski J, Berg RA, Tijssen JG, Koster RW (2010). Global incidences of out-of-hospital cardiac arrest and survival rates: systematic review of 67 prospective studies. Resuscitation.

[15] Bobrow BJ, Vadeboncoeur TF, Spaite DW (2011). The effectiveness of ultrabrief and brief educational videos for training lay responders in hands-only cardiopulmonary resuscitation: implications for the future of citizen cardiopulmonary resuscitation training. Circ Cardiovasc Qual Outcomes.

[16] Shaik Alavudeen S, Basharat V, Khaled Bahamdan A (2022). Knowledge, attitude and preparedness of healthcare students toward basic life support at King Khalid University, Abha, Kingdom of Saudi Arabia. Clin Exp Hypertens.

[17] Lu C, Jin Y, Meng F (2016). An exploration of attitudes toward bystander cardiopulmonary resuscitation in university students in Tianjin, China: a survey. Int Emerg Nurs.

[18] Frei HC, Hotz T, Cadosch D, Rudin M, Käch K (2012). Central head perforation, or "cut through," caused by the helical blade of the proximal femoral nail antirotation. J Orthop Trauma.

[19] Bray JE, Smith K, Case R, Cartledge S, Straney L, Finn J (2017). Public cardiopulmonary resuscitation training rates and awareness of hands-only cardiopulmonary resuscitation: a cross-sectional survey of Victorians. Emerg Med Australas.

[20] Coons SJ, Guy MC (2009). Performing bystander CPR for sudden cardiac arrest: behavioral intentions among the general adult population in Arizona. Resuscitation.

[21] Kleinman ME, Brennan EE, Goldberger ZD (2015). Part 5: adult basic life support and cardiopulmonary resuscitation quality: 2015 American Heart Association guidelines update for cardiopulmonary resuscitation and emergency cardiovascular care. Circulation.

[22] Savastano S, Vanni V (2011). Cardiopulmonary resuscitation in real life: the most frequent fears of lay rescuers. Resuscitation.

[23] Malta Hansen C, Rosenkranz SM, Folke F (2017). Lay bystanders' perspectives on what facilitates cardiopulmonary resuscitation and use of automated external defibrillators in real cardiac arrests. J Am Heart Assoc.

